# Supplementary material for: A broader lens on tuberculosis cost-effectiveness analysis: How patient-incurred costs and post-tuberculosis outcomes reshape estimates in a multi-country study
Source: PLOS Glob Public Health. 2025 Sep 11;5(9):e0005062. doi: 10.1371/journal.pgph.0005062 (PMC12425205; doi:10.1371/journal.pgph.0005062)
Supplement: S1 Text — (PDF) [file pgph.0005062.s001.pdf]

S1 Text: TABLES and additional Supporting Info: A broader lens on tuberculosis cost-effectiveness analysis: How patient-incurred costs and post-tuberculosis outcomes reshape estimates in a multi-country study

Ewan M. Tomeny*[1], Phuong Bich Tran[2], Joseph Kazibwe[3], Laura Rosu[1], Georgios F. Nikolaidis[4], Rebecca Nightingale[1,5], Tom Wingfield[1,6,7], Jamilah Meghji[8], S. Bertel Squire[1,7], and Eve Worrall[1]

[1]Centre for Tuberculosis Research, Departments of Clinical Sciences and International Public Health, Liverpool School of Tropical Medicine, Liverpool, United Kingdom

[2]Nuffield Department of Primary Care Health Sciences, University of Oxford, Oxford, United Kingdom

[3]Department of Clinical Sciences, Lund University, Malmö, Sweden

[4]Methods and Evidence Generation Department, Centre of Excellence in Evidence Synthesis, IQVIA Ltd, London, United Kingdom

[5]Respiratory Department, Liverpool University Hospitals NHS Foundation Trust, Liverpool, United Kingdom

[6]Department of Global Public Health, Karolinska Institutet, Stockholm, Sweden

[7]Tropical and Infectious Diseases Unit, Liverpool University Hospitals NHS Foundation Trust, Liverpool, United Kingdom

[8]National Heart & Lung Institute, Imperial College London, London, United Kingdom

* ewan.tomeny@lstmed.ac.uk

Table A in S1 Text: Estimated number of Cases, Deaths and DALYs under Conventional and Extended Approaches, for a population of 100,000 receiving the intervention

	**Without Intervention**								**With Intervention**								**Difference**							
Country	(Independent of timeframe)				Conventional†		Extended†		(Independent of timeframe)				Conventional†		Extended†		(Independent of timeframe)				Conventional†		Extended†	
	Cases	Trtd	Die	YLL	YLD	DALYs	YLD	DALYs	Cases	Trtd	Die	YLL	YLD	DALYs	YLD	DALYs	Cases	Trtd	Die	YLL	YLD	DALYs	YLD	DALYs
Brazil	48.5	40.3	5.2	124.4	10	134.3	55.6	180	17.5	11.9	1.9	44.8	4.6	49.4	20.4	65.2	31.1	28.4	3.3	79.6	5.4	85	35.2	114.8
Burkina Faso	44	33	6	170.3	9.3	179.6	30.8	201.1	15.8	11.2	2.2	61.3	5.4	66.7	8.7	70	28.2	21.8	3.8	109	4	112.9	22.1	131.1
DRC	317	247.3	40	1055.4	66	1121.4	370.2	1425.6	114.1	83	14.4	379.9	38.5	418.4	147	526.9	202.9	164.3	25.6	675.5	27.5	703	223.2	898.7
Fiji	66.4	61.7	5.3	104.9	12.1	117	80.8	185.7	23.9	20.6	1.9	37.7	6.2	43.9	30.6	68.4	42.5	41.1	3.4	67.1	5.9	73	50.2	117.3
Ghana	132.3	49	42	945.7	37.4	983.1	160	1105.8	47.6	15.2	15.1	340.5	22.9	363.3	66	406.5	84.7	33.8	26.9	605.3	14.5	619.8	94	699.3
Indonesia	385.1	258	51	994.9	87	1081.9	539.7	1534.6	138.6	90.8	18.4	358.2	55.8	414	218.3	576.5	246.5	167.3	32.6	636.7	31.1	667.9	321.4	958.2
Kenya	236.5	163.2	49	1106.9	54.6	1161.6	253.5	1360.5	85.2	44.7	17.6	398.5	25.4	423.9	93.6	492.1	151.4	118.6	31.4	708.4	29.2	737.7	160	868.4
Laos	136.8	112.2	15	363.8	27.3	391	130.6	494.3	49.3	38.6	5.4	131	15.6	146.6	52.1	183	87.6	73.6	9.6	232.8	11.6	244.4	78.5	311.3
Mali	48.9	34.7	7.7	205.1	10.8	215.8	41.6	246.6	17.6	11.4	2.8	73.8	6.2	80	14.6	88.4	31.3	23.3	4.9	131.2	4.6	135.8	27	158.3
Mongolia	450.5	81.1	13	412.6	141.5	554.2	617.2	1029.8	162.2	29.2	4.7	148.5	102.8	251.4	274.1	422.6	288.3	51.9	8.3	264.1	38.7	302.8	343.1	607.2
Myanmar	471.8	217	91	1893.3	125.6	2018.9	633.6	2526.9	169.8	73.4	32.8	681.6	81	762.6	262.3	943.9	301.9	143.6	58.2	1211.7	44.5	1256.3	371.3	1583
Nigeria	220.1	129.9	45	1113.5	53	1166.5	254.3	1367.8	79.2	44.1	16.2	400.9	33.3	434.1	105	505.9	140.9	85.8	28.8	712.6	19.7	732.3	149.3	862
PNG	430.7	344.5	50	1436.8	89.3	1526.1	712	2148.8	155	117.2	18	517.2	52.2	569.4	274.8	792	275.6	227.3	32	919.5	37.1	956.7	437.2	1356.7
Philippines	636.9	375.8	35	825.3	153.3	978.6	808.7	1634.1	229.3	132.7	12.6	297.1	97.8	394.9	332.8	630	407.6	243.1	22.4	528.2	55.5	583.7	475.9	1004.1
Solomon Isls	59	47.2	6.5	184.6	11.9	196.5	89.2	273.8	21.2	17	2.3	66.5	7.7	74.2	35.5	102	37.7	30.2	4.2	118.2	4.2	122.4	53.6	171.8
Tanzania	195.9	152.8	27	723.4	41.6	765	232.5	956	70.5	45.7	9.7	260.4	21	281.4	87.9	348.3	125.4	107.1	17.3	463	20.6	483.6	144.7	607.7
Thailand	155.5	101.1	19	319.4	36	355.4	179.1	498.5	56	33.4	6.8	115	20.9	135.9	71.5	186.5	99.5	67.7	12.2	204.4	15.1	219.5	107.6	312
Uganda	197.3	197.3	9.9	237.2	35.5	272.7	190.4	427.6	71	47.6	3.6	85.4	11.8	97.2	64.4	149.8	126.2	149.7	6.3	151.8	23.7	175.4	126	277.8
Viet Nam	176.4	104.1	14	250.2	42.7	292.9	191.9	442.1	63.5	36.5	5	90.1	26.8	116.8	80.2	170.3	112.9	67.5	9	160.1	16	176.1	111.7	271.8

† The ‘Conventional’ time period does not consider disability in the post-TB period, while the ‘Extended’ time period does include the post-TB period. Data in this table are not affected by change of perspective (as contain no costs).

Table B in S1 Text: Estimates for TB Prevalence, HIV Co-infection Rates, and Mortality Data Across Study Countries (WHO 2022 data)

Country*	Population (thousands)	TB Incidence (/100k)		Proportion TB which is HIV+		Mortality among HIV- (/100k)		Mortality among HIV+ (/100k)		Case Detection Rate‡		Proportion TB MDR-TB	
Brazil	215,313	49	[42, 56]	0.18	[0.14, 0.23]	3.4	[3.2, 3.5]	1.8	[1.2, 2.6]	0.83	[0.72, 0.98]	0.03	[0, 0.06]
Burkina Faso	22,674	44	[28, 64]	0.055	[0.05, 0.061]	5.4	[3.1, 8.2]	0.64	[0.4, 0.92]	0.75	[0.52, 1.2]	0.01	[0.01, 0.03]
DRC	99,010	317	[205, 454]	0.068	[0.067, 0.069]	34	[20, 52]	5.2	[3.4, 7.4]	0.78	[0.55, 1.2]	0.02	[0, 0.05]
Fiji	930	66	[51, 84]	0.072	[0.051, 0.095]	4.4	[4.3, 4.5]	0.88	[0.51, 1.3]	0.93	[0.73, 1.2]	0.02	[0, 0.04]
Ghana	33,476	133	[60, 234]	0.14	[0.13, 0.14]	32	[14, 58]	9.6	[4.4, 17]	0.37	[0.21, 0.82]	0.02	[0, 0.08]
Indonesia	275,501	385	[335, 423]	0.023	[0.01, 0.041]	49	[43, 55]	2.4	[2.2, 2.7]	0.67	[0.61, 0.77]	0.03	[0.02, 0.05]
Kenya	54,027	237	[149, 363]	0.24	[0.11, 0.4]	32	[17, 52]	16	[9.8, 25]	0.69	[0.45, 1.1]	0.01	[0, 0.03]
Laos	7,529	138	[86, 202]	0.045	[0.041, 0.05]	13	[7.5, 21]	1.3	[0.82, 1.9]	0.82	[0.56, 1.3]	0.01	[0, 0.02]
Mali	22,594	49	[31, 71]	0.088	[0.082, 0.094]	6.5	[3.8, 9.8]	1.2	[0.79, 1.8]	0.71	[0.49, 1.1]	0.01	[0, 0.04]
Mongolia	3,398	452	[252, 799]	0	[0, 0.0007]	12	[11, 14]	0.33	[0.29, 0.37]	0.18	[0.1, 0.33]	0.07	[0.02, 0.22]
Myanmar	54,179	475	[312, 675]	0.06	[0.059, 0.061]	80	[55, 110]	11	[7.5, 15]	0.46	[0.32, 0.7]	0.05	[0.02, 0.11]
Nigeria	218,541	219	[143, 311]	0.057	[0.056, 0.058]	40	[24, 60]	4.7	[3.1, 6.6]	0.59	[0.42, 0.9]	0.03	[0.01, 0.05]
PNG	10143	432	[348, 525]	0.055	[0.053, 0.058]	44	[29, 63]	5.4	[3.9, 7.1]	0.8	[0.66, 1]	0.05	[0.01, 0.11]
Philippines	115,559	638	[337, 1060]	0.019	[0.011, 0.03]	34	[31, 37]	0.69	[0.63, 0.75]	0.59	[0.36, 1.1]	0.04	[0.01, 0.13]
Solomon Islands†	724	59	[45, 75]	-	-	6.5	[4.2, 9.3]	-	-	0.8	[0.63, 1]	0.02	[0, 0.05]
Tanzania	71,697	195	[81, 358]	0.083	[0.052, 0.12]	19	[8.3, 34]	7.8	[3.8, 13]	0.78	[0.43, 1.9]	0.02	[0.01, 0.04]
Thailand	47,250	155	[122, 193]	0.33	[0.32, 0.33]	16	[13, 20]	2.9	[2, 4]	0.65	[0.52, 0.83]	0.01	[0, 0.04]
Uganda	65,498	198	[119, 297]	0.17	[0.16, 0.17]	4	[0.62, 11]	5.9	[2.1, 12]	1	[0.67, 1.7]	0.01	[0, 0.05]
Viet Nam	98,187	176	[121, 251]	0.025	[0.024, 0.026]	11	[7.7, 15]	2.7	[1.8, 3.6]	0.59	[0.42, 0.86]	0.05	[0.02, 0.11]

* DRC: Democratic Republic of the Congo; PNG: Papua New Guinea. All data are 2022 estimates taken from WHO database . [?]

† HIV prevalence in Solomon Islands very low (∼0.002%); data are for HIV-negative TB patients only.

‡ The case detection rate, (or 'TB treatment coverage'), is the percentage of people with active TB who receive treatment.

Table C in S1 Text: Defining variables

**Cost**		
Incremental Total Health System Costs	ΔC_{hs}	The incremental cost incurred by the health system for implementing an intervention.
Incremental Individual Health System Cost	$\Delta C_{\text{hs,ind}}$	The incremental cost incurred by the health system for implementing an intervention per individual.
Incremental TB Patient Cost	$\Delta C_{\text{p,ind}}$	The incremental costs (direct and indirect) borne by an individual patient as a result of TB treatment or management.
Incremental Total Patient Costs	$\Delta C_{\text{pat}} = \sum \Delta C_{\text{pat,ind}}$	The sum of all incremental TB patient costs.
Incremental Total Societal Costs	$\Delta C_{\text{soc}} = \Delta C_{\text{hs}} + \Delta C_{\text{pat}}$	The combined incremental total of health system and patient costs.
**Effectiveness**		
Incremental DALYs due to YLL	ΔD_{YLL}	The incremental number of DALYs attributed to years of life lost (i.e., mortality).
Incremental DALYs due to active TB disease	ΔD_{aTB}	The incremental number of DALYs attributed to active TB disease.
Incremental DALYs due to ltd	ΔD_{ltd}	The incremental number of DALYs attributed to post-TB lung disease.
Incremental DALYs due to treatment/intervention	ΔD_{Tx}	The incremental number of DALYs attributed to anti-TB treatment.
Incremental DALYs due to YLD	$\Delta D_{\text{YLD}} = \Delta D_{\text{aTB}} + \Delta D_{\text{ltd}} + \Delta D_{\text{Tx}}$	The incremental number of DALYs attributed to years lived with disability (i.e., morbidity).
Incremental Total DALYs	$\Delta D = \Delta D_{\text{YLL}} + \Delta D_{\text{YLD}}$	The incremental total number of disability-adjusted life years.
**Diagnostic Accuracy and PPV**		
Positive Predictive Value (PPV)	$PPV = \frac{TP}{TP+FP}$	The proportion of true positive results in all positive test results.
Number of True Positives	TP	The number of individuals correctly identified as having the disease.
Number of False Positives	FP	The number of individuals incorrectly identified as having the disease.
**Cost-Effectiveness Analysis**		
ICER	$\frac{\Delta C_{\text{soc}}}{\Delta D}$	Incremental Cost-effectiveness Ratio: The ratio of the incremental difference in total societal costs to the incremental difference in effectiveness (total DALYs averted) between an intervention and a comparator.
Cost-effectiveness Threshold	k	The maximum cost society is willing to pay to avert one DALY, distinguishing between cost-effective and non-cost-effective interventions.
**Analysis of Cost Distribution**		
Ratio of individual Health System to individual 'Societal' Costs (β)	$\beta = \frac{\Delta C_{\text{hs,ind}}}{\Delta C_{\text{hs,ind}} + \Delta C_{\text{pat,ind}}}$	The proportion of the health system's cost per individual to the total of individual health system and patient costs, reflecting the share of health system expenditures within the overall economic impact of an intervention.

Model equations

DALY calculations were stratified by age group (i) and TB type (j), distinguishing drug-susceptible and multidrug-resistant TB. YLLs were further stratified by treatment status (k), to reflect differences in mortality among treated and untreated individuals. YLDs included morbidity during active disease (covering pre-treatment and treatment periods) and long-term post-TB morbidity. Disability weights were fixed across age groups and TB types, but time spent in each state varied by TB type. Costs were estimated only for individuals who received treatment, and stratified by TB type (j), but applied uniformly across age groups. All calculations were performed separately for each country.

$$
\begin{aligned}
YLL_{ijk} &= N_{ijk} \cdot CFR_{jk} \cdot LE_i \\
YLD_{ij}^{\text{active}} &= N_{ij} \cdot DW_{\text{aTB}} \cdot (T_j^{\text{preTx}} + T_j^{\text{Tx}}) \\
YLD_{ij}^{\text{postTB}} &= N_{ij} \cdot DW_{\text{ltd}} \cdot T^{\text{postTB}} \\
DALY_{ij} &= \sum_k YLL_{ijk} + YLD_{ij}^{\text{active}} + YLD_{ij}^{\text{postTB}}
\end{aligned}
$$

Where:

- N_{ijk} = Number of TB cases in age group i, TB type j, treatment status k
- N_{ij}^{treated} = Number of treated TB cases in age group i, TB type j
- CFR_{jk} = Case fatality rate by TB type and treatment status
- LE_i = Life expectancy at age i
- DW_{aTB} = Disability weight for active TB
- DW_{ltd} = Disability weight for post-TB morbidity (long-term disability)
- T_j^{preTx} = Pre-treatment duration for TB type j
- T_j^{Tx} = Treatment duration for TB type j
- T^{postTB} = Post-TB morbidity duration

Lost Income Post-Treatment Completion

From National TB patient cost surveys, we took the monthly income pre-TB (I_{before}) and the monthly income during treatment (I_{during}). These values were converted to 2022 USD, and the difference between these two values (δ_{mon}) represents the monthly lost income.

Linear Recovery Over t Years

- Initial monthly loss: $\delta_{\text{mon}} = I_{\text{before}} - I_{\text{during}}$
- Recovery period: t years ($12 \times t$ months)

Total Lost Income Calculation

$$\text{Total cost over } t \text{ years} = \frac{\delta_{\text{mon}} \times (t \times 12)}{2}$$

As a linear return, the assumed lost amount each year is constant, equal to $\delta_{\text{mon}} \times 6$.

Table D in S1 Text: Total household patient costs by years following diagnosis (assuming linear return to pre-TB level)

		Years for household finances to recover to pre-TB level									
		1	2	3	4	5	6	7	8	9	10
Country	δ_{mon}	Total household patient cost over years (USD)									
Brazil	91	545	1090	1635	2181	2726	3271	3816	4361	4906	5452
Burkina Faso	98	588	1175	1763	2351	2938	3526	4113	4701	5289	5876
DRC	30	179	359	538	717	896	1076	1255	1434	1614	1793
Fiji	21	123	246	369	492	616	739	862	985	1108	1231
Ghana	247	1483	2966	4450	5933	7416	8899	10382	11866	13349	14832
Indonesia	28	165	331	496	661	827	992	1158	1323	1488	1654
Kenya	106	638	1277	1915	2554	3192	3830	4469	5107	5746	6384
Laos	85	509	1017	1526	2035	2544	3052	3561	4070	4579	5087
Mali	210	1261	2521	3782	5042	6303	7564	8824	10085	11345	12606
Mongolia	164	985	1971	2956	3941	4927	5912	6897	7883	8868	9853
Myanmar	119	716	1432	2148	2864	3581	4297	5013	5729	6445	7161
Nigeria	132	792	1584	2376	3168	3960	4752	5544	6336	7127	7919
Papua New Guinea	0	0	0	0	0	0	0	0	0	0	0
Philippines	40	240	479	719	958	1198	1437	1677	1917	2156	2396
Solomon Islands	15	92	183	275	366	458	549	641	732	824	916
Tanzania	0	0	0	0	0	0	0	0	0	0	0
Thailand	278	1666	3333	4999	6665	8332	9998	11664	13331	14997	16663
Uganda	68	406	813	1219	1625	2031	2438	2844	3250	3656	4063
Viet Nam	115	693	1385	2078	2770	3463	4155	4848	5541	6233	6926

In both Papua New Guinea and Tanazania average household income was recorded as higher during TB than prior, so calculation based on monthly income loss was not possible.

When introducing an annual discount rate of 3% (varied in SA), each year's total losses were first calculated and discounted, and then summed for t years using:

$$\text{Total cost over } t \text{ years} = \sum_{i=1}^{t} \left(\frac{\delta_{\text{mon}} \times 6}{(1.03)^i} \right)$$

where t is the number of years and i is each individual year.

Table E in S1 Text: Total household patient costs by years following diagnosis (assuming linear return to pre-TB level), applying a 3% annual discount rate

		Years for household finances to recover to pre-TB level									
		1	2	3	4	5	6	7	8	9	10
Country	δ_{mon}	Total household patient cost over years (USD)									
Brazil	91	529	1043	1542	2026	2497	2953	3397	3827	4245	4650
Burkina Faso	98	571	1124	1662	2184	2691	3183	3661	4125	4575	5013
DRC	30	174	343	507	666	821	971	1117	1258	1396	1529
Fiji	21	120	236	348	458	564	667	767	864	959	1050
Ghana	247	1440	2838	4195	5513	6793	8035	9241	10412	11548	12652
Indonesia	28	161	316	468	615	757	896	1030	1161	1288	1411
Kenya	106	620	1222	1806	2373	2924	3458	3977	4481	4971	5446
Laos	85	494	973	1439	1891	2330	2756	3170	3571	3961	4340
Mali	210	1224	2412	3566	4686	5773	6829	7854	8849	9815	10753
Mongolia	164	957	1885	2787	3663	4512	5338	6139	6917	7672	8405
Myanmar	119	695	1370	2026	2662	3280	3879	4462	5027	5576	6108
Nigeria	132	769	1515	2240	2944	3627	4290	4934	5559	6166	6755
Papua New Guinea	0	0	0	0	0	0	0	0	0	0	0
Philippines	40	233	458	678	891	1097	1298	1493	1682	1865	2044
Solomon Islands	15	89	175	259	340	419	496	570	643	713	781
Tanzania	0	0	0	0	0	0	0	0	0	0	0
Thailand	278	1618	3188	4713	6194	7631	9027	10382	11697	12974	14214
Uganda	68	394	777	1149	1510	1861	2201	2531	2852	3163	3466
Viet Nam	115	672	1325	1959	2574	3172	3752	4315	4862	5393	5908

Table F in S1 Text: Cost to the intervention recipient that make the *Health System Perspective Extended* ICER or *Societal Perspective Conventional* ICER appear more cost-effective than the Extended Societal ICER

Country	ICER HS Extended	ICER HS Standard
Brazil	$1	$6
Burkina Faso	$1	$1
DRC	$2	$4
Fiji	$1	$5
Ghana	$2	$5
Indonesia	$2	$24
Kenya	$3	$7
Laos	$3	$5
Mali	$2	$2
Mongolia	$3	$27
Myanmar	$6	$12
Nigeria	$4	$15
Papua New Guinea	$1	$17
Philippines	$4	$28
Solomon Islands	$1	$3
Tanzania	$1	$3
Thailand	$5	$15
Uganda	$2	$3
Viet Nam	$3	$9

As an example, for Mongolia, if the average cost incurred by recipients of the preventative intervention, for example the opportunity cost of lost income from travelling to receive a vaccine, were $3 or more, then the ICER for the intervention under the health system perspective (not including patient costs) would be more favourable (lower) than under the Health system perspective. That is, overall patient costs would be negative.

Table G in S1 Text: Scenario analyses results of varying active TB disability weight for active TB [0.224-0.454]. Percentage change of ICERs relative to Extended evaluation, Societal Perspective

Country	Health system perspective Extended	Societal perspective Conventional	Health system perspective Conventional
Brazil	[0.06%, -0.06%]	[0.55%, -0.59%]	[0.58%, -0.62%]
Burkina Faso	[0.3%, -0.32%]	[0.5%, -0.54%]	[0.52%, -0.56%]
DRC	[0.22%, -0.24%]	[0.53%, -0.57%]	[0.63%, -0.67%]
Fiji	[0.07%, -0.08%]	[1.27%, -1.32%]	[1.31%, -1.36%]
Ghana	[0.07%, -0.07%]	[0.17%, -0.19%]	[0.19%, -0.21%]
Indonesia	[0.04%, -0.04%]	[0.85%, -0.89%]	[0.86%, -0.91%]
Kenya	[0.06%, -0.07%]	[0.22%, -0.24%]	[0.23%, -0.25%]
Laos	[0.34%, -0.36%]	[0.68%, -0.72%]	[0.83%, -0.88%]
Mali	[0.46%, -0.49%]	[0.61%, -0.65%]	[0.67%, -0.72%]
Mongolia	[0.29%, -0.3%]	[5.64%, -5.1%]	[5.75%, -5.2%]
Myanmar	[0.22%, -0.24%]	[0.57%, -0.6%]	[0.64%, -0.68%]
Nigeria	[0.12%, -0.13%]	[0.3%, -0.32%]	[0.34%, -0.37%]
Papua New Guinea	[0.01%, -0.01%]	[0.55%, -0.59%]	[0.56%, -0.6%]
Philippines	[0.19%, -0.19%]	[2.49%, -2.47%]	[2.57%, -2.55%]
Solomon Islands	[0.1%, -0.11%]	[0.62%, -0.66%]	[0.7%, -0.75%]
Tanzania	[0.03%, -0.03%]	[0.3%, -0.32%]	[0.33%, -0.35%]
Thailand	[0.24%, -0.26%]	[1.17%, -1.21%]	[1.19%, -1.24%]
Uganda	[0.45%, -0.48%]	[1.34%, -1.41%]	[1.48%, -1.55%]
Viet Nam	[0.46%, -0.48%]	[2.11%, -2.11%]	[2.3%, -2.3%]

Table H in S1 Text: Scenario analyses results of varying time for financial recovery [1-10 years]. Percentage change of ICERs relative to Extended evaluation, Societal Perspective

Country	Health system perspective Extended	Societal perspective Conventional	Health system perspective Conventional
Brazil	[-1.15%, 4.02%]	[-1.17%, 4.08%]	[-1.15%, 4.02%]
Burkina Faso	[-9.58%, 33.54%]	[-9.75%, 34.12%]	[-9.58%, 33.54%]
DRC	[-4.11%, 14.4%]	[-4.44%, 15.54%]	[-4.11%, 14.4%]
Fiji	[-0.76%, 2.67%]	[-0.78%, 2.72%]	[-0.76%, 2.67%]
Ghana	[-2.65%, 9.28%]	[-2.7%, 9.46%]	[-2.65%, 9.28%]
Indonesia	[-0.64%, 2.24%]	[-0.64%, 2.26%]	[-0.64%, 2.24%]
Kenya	[-2.74%, 9.59%]	[-2.76%, 9.67%]	[-2.74%, 9.59%]
Laos	[-4.97%, 17.4%]	[-5.48%, 19.19%]	[-4.97%, 17.4%]
Mali	[-13.89%, 48.6%]	[-15%, 52.51%]	[-13.89%, 48.6%]
Mongolia	[-1.8%, 6.31%]	[-1.84%, 6.45%]	[-1.8%, 6.31%]
Myanmar	[-4.64%, 16.25%]	[-4.88%, 17.09%]	[-4.64%, 16.25%]
Nigeria	[-3.3%, 11.56%]	[-3.43%, 11.99%]	[-3.3%, 11.56%]
Papua New Guinea	[0%, 0%]	[0%, 0%]	[0%, 0%]
Philippines	[-1.65%, 5.76%]	[-1.69%, 5.91%]	[-1.65%, 5.76%]
Solomon Islands	[-0.76%, 2.67%]	[-0.81%, 2.83%]	[-0.76%, 2.67%]
Tanzania	[0%, 0%]	[0%, 0%]	[0%, 0%]
Thailand	[-4.75%, 16.64%]	[-4.82%, 16.85%]	[-4.75%, 16.64%]
Uganda	[-8.3%, 29.05%]	[-9.18%, 32.11%]	[-8.3%, 29.05%]
Viet Nam	[-3.92%, 13.71%]	[-4.17%, 14.61%]	[-3.92%, 13.71%]

Table I in S1 Text: Scenario analyses results of using a uniform life-long post-TB disability weight of 0.053. Percentage change of ICERs relative to Extended evaluation, Societal Perspective

Country	Health system perspective Extended	Societal perspective Conventional	Health system perspective Conventional
Brazil	-0.7%	+26.39%	+25.97
Burkina Faso	-1.81%	+10.22%	+9.95
DRC	-1.78%	+13.87%	+12.72
Fiji	-0.78%	+30.87%	+30.23
Ghana	-0.46%	+8.05%	+7.9
Indonesia	-0.27%	+18.89%	+18.77
Kenya	-0.68%	+15.02%	+14.93
Laos	-2.51%	+15.74%	+14.26
Mali	-3.14%	+8.88%	+8.09
Mongolia	-1.46%	+42.53%	+41.62
Myanmar	-1.51%	+13.72%	+13.09
Nigeria	-0.95%	+11.83%	+11.39
Papua New Guinea	-0.11%	+18.18%	+18.07
Philippines	-1.36%	+37.61%	+36.7
Solomon Islands	-0.85%	+14.75%	+13.93
Tanzania	-0.31%	+16.39%	+15.99
Thailand	-1.84%	+25.93%	+25.6
Uganda	-5.96%	+30.81%	+27.72
Viet Nam	-2.9%	+29.33%	+27.49

Table J in S1 Text: Three-way sensitivity analysis for SAE parameters. Table presents percentage increase in ICER for Brazil for ICER for Extended evaluation under a Societal Perspective

Duration of SAE (years)	Rate of SAE →										Disability weight 0.1										
↓	0.01%	0.05%	0.10%	0.15%	0.20%	0.25%	0.30%	0.35%	0.40%	0.45%	0.50%	0.55%	0.60%	0.65%	0.70%	0.75%	0.80%	0.85%	0.90%	0.95%	1.00%
0.02	0%	0%	0%	0%	0%	0%	0%	0%	0%	1%	1%	1%	1%	1%	1%	1%	1%	1%	1%	1%	1%
0.04	0%	0%	0%	0%	0%	1%	1%	1%	1%	1%	1%	1%	1%	1%	2%	2%	2%	2%	2%	2%	2%
0.06	0%	0%	0%	1%	1%	1%	1%	1%	1%	2%	2%	2%	2%	2%	2%	3%	3%	3%	3%	3%	3%
0.08	0%	0%	0%	1%	1%	1%	1%	2%	2%	2%	2%	3%	3%	3%	3%	3%	4%	4%	4%	4%	5%
0.1	0%	0%	1%	1%	1%	1%	2%	2%	2%	3%	3%	3%	3%	4%	4%	4%	5%	5%	5%	6%	6%
0.12	0%	0%	1%	1%	1%	2%	2%	2%	3%	3%	3%	4%	4%	5%	5%	5%	6%	6%	6%	7%	7%
0.14	0%	0%	1%	1%	2%	2%	2%	3%	3%	4%	4%	4%	5%	5%	6%	6%	7%	7%	8%	8%	8%
0.16	0%	0%	1%	1%	2%	2%	3%	3%	4%	4%	5%	5%	6%	6%	7%	7%	8%	8%	9%	9%	10%
0.18	0%	1%	1%	2%	2%	3%	3%	4%	4%	5%	5%	6%	6%	7%	8%	8%	9%	9%	10%	11%	11%
0.2	0%	1%	1%	2%	2%	3%	3%	4%	5%	5%	6%	7%	7%	8%	8%	9%	10%	10%	11%	12%	13%
0.22	0%	1%	1%	2%	3%	3%	4%	4%	5%	6%	7%	7%	8%	9%	9%	10%	11%	12%	12%	13%	14%
0.24	0%	1%	1%	2%	3%	3%	4%	5%	6%	6%	7%	8%	9%	10%	10%	11%	12%	13%	14%	15%	15%
0.26	0%	1%	1%	2%	3%	4%	5%	5%	6%	7%	8%	9%	10%	10%	11%	12%	13%	14%	15%	16%	17%
0.28	0%	1%	2%	2%	3%	4%	5%	6%	7%	8%	8%	9%	10%	11%	12%	13%	14%	15%	16%	17%	18%
0.3	0%	1%	2%	3%	3%	4%	5%	6%	7%	8%	9%	10%	11%	12%	13%	14%	15%	17%	18%	19%	20%
0.32	0%	1%	2%	3%	4%	5%	6%	7%	8%	9%	10%	11%	12%	13%	14%	15%	17%	18%	19%	20%	22%
0.34	0%	1%	2%	3%	4%	5%	6%	7%	8%	9%	10%	12%	13%	14%	15%	17%	18%	19%	21%	22%	23%
0.36	0%	1%	2%	3%	4%	5%	6%	8%	9%	10%	11%	12%	14%	15%	16%	18%	19%	21%	22%	24%	25%
0.38	0%	1%	2%	3%	4%	6%	7%	8%	9%	11%	12%	13%	15%	16%	17%	19%	20%	22%	24%	25%	27%
0.4	0%	1%	2%	3%	5%	6%	7%	8%	10%	11%	13%	14%	15%	17%	18%	20%	22%	23%	25%	27%	29%
0.42	0%	1%	2%	4%	5%	6%	8%	9%	10%	12%	13%	15%	16%	18%	20%	21%	23%	25%	27%	29%	31%
0.44	0%	1%	3%	4%	5%	7%	8%	9%	11%	12%	14%	16%	17%	19%	21%	23%	24%	26%	28%	30%	33%
0.46	0%	1%	3%	4%	5%	7%	8%	10%	11%	13%	15%	16%	18%	20%	22%	24%	26%	28%	30%	32%	34%
0.48	0%	1%	3%	4%	6%	7%	9%	10%	12%	14%	15%	17%	19%	21%	23%	25%	27%	29%	32%	34%	37%
0.5	0%	1%	3%	4%	6%	7%	9%	11%	13%	14%	16%	18%	20%	22%	24%	26%	29%	31%	33%	36%	39%

Duration of SAE (years)	Rate of SAE →										Disability weight 0.2										
↓	0.01%	0.05%	0.10%	0.15%	0.20%	0.25%	0.30%	0.35%	0.40%	0.45%	0.50%	0.55%	0.60%	0.65%	0.70%	0.75%	0.80%	0.85%	0.90%	0.95%	1.00%
0.02	0%	0%	0%	0%	0%	1%	1%	1%	1%	1%	1%	1%	1%	1%	2%	2%	2%	2%	2%	2%	2%
0.04	0%	0%	0%	1%	1%	1%	1%	2%	2%	2%	2%	3%	3%	3%	3%	3%	4%	4%	4%	4%	5%
0.06	0%	0%	1%	1%	1%	2%	2%	2%	3%	3%	3%	4%	4%	5%	5%	5%	6%	6%	6%	7%	7%
0.08	0%	0%	1%	1%	2%	2%	3%	3%	4%	4%	5%	5%	6%	6%	7%	7%	8%	8%	9%	9%	10%
0.1	0%	1%	1%	2%	2%	3%	3%	4%	5%	5%	6%	7%	7%	8%	8%	9%	10%	10%	11%	12%	13%
0.12	0%	1%	1%	2%	3%	3%	4%	5%	6%	6%	7%	8%	9%	10%	10%	11%	12%	13%	14%	15%	15%
0.14	0%	1%	2%	2%	3%	4%	5%	6%	7%	8%	8%	9%	10%	11%	12%	13%	14%	15%	16%	17%	18%
0.16	0%	1%	2%	3%	4%	5%	6%	7%	8%	9%	10%	11%	12%	13%	14%	15%	17%	18%	19%	20%	22%
0.18	0%	1%	2%	3%	4%	5%	6%	8%	9%	10%	11%	12%	14%	15%	16%	18%	19%	21%	22%	24%	25%
0.2	0%	1%	2%	3%	5%	6%	7%	8%	10%	11%	13%	14%	15%	17%	18%	20%	22%	23%	25%	27%	29%
0.22	0%	1%	3%	4%	5%	7%	8%	9%	11%	12%	14%	16%	17%	19%	21%	23%	24%	26%	28%	30%	33%
0.24	0%	1%	3%	4%	6%	7%	9%	10%	12%	14%	15%	17%	19%	21%	23%	25%	27%	29%	32%	34%	37%
0.26	0%	1%	3%	5%	6%	8%	10%	11%	13%	15%	17%	19%	21%	23%	25%	28%	30%	33%	35%	38%	41%
0.28	0%	2%	3%	5%	7%	8%	10%	12%	14%	16%	18%	21%	23%	25%	28%	31%	33%	36%	39%	42%	45%
0.3	0%	2%	3%	5%	7%	9%	11%	13%	15%	18%	20%	23%	25%	28%	31%	33%	37%	40%	43%	47%	50%
0.32	0%	2%	4%	6%	8%	10%	12%	14%	17%	19%	22%	24%	27%	30%	33%	37%	40%	44%	47%	51%	55%
0.34	0%	2%	4%	6%	8%	10%	13%	15%	18%	21%	23%	26%	29%	33%	36%	40%	44%	48%	52%	56%	61%
0.36	0%	2%	4%	6%	9%	11%	14%	16%	19%	22%	25%	28%	32%	35%	39%	43%	47%	52%	57%	62%	67%
0.38	0%	2%	4%	7%	9%	12%	15%	17%	20%	24%	27%	30%	34%	38%	42%	47%	51%	56%	62%	67%	74%
0.4	0%	2%	5%	7%	10%	13%	15%	18%	22%	25%	29%	33%	37%	41%	45%	50%	55%	61%	67%	74%	80%
0.42	0%	2%	5%	8%	10%	13%	16%	20%	23%	27%	31%	35%	39%	44%	49%	54%	60%	66%	73%	80%	88%
0.44	0%	3%	5%	8%	11%	14%	17%	21%	24%	28%	33%	37%	42%	47%	52%	58%	65%	72%	79%	87%	96%
0.46	1%	3%	5%	8%	11%	15%	18%	22%	26%	30%	34%	39%	44%	50%	56%	63%	70%	77%	86%	95%	105%
0.48	1%	3%	6%	9%	12%	15%	19%	23%	27%	32%	37%	42%	47%	53%	60%	67%	75%	83%	93%	103%	115%
0.5	1%	3%	6%	9%	13%	16%	20%	24%	29%	33%	39%	44%	50%	57%	64%	72%	80%	90%	101%	113%	126%

Duration of SAE (years)	Rate of SAE →										Disability weight 0.3										
↓	0.01%	0.05%	0.10%	0.15%	0.20%	0.25%	0.30%	0.35%	0.40%	0.45%	0.50%	0.55%	0.60%	0.65%	0.70%	0.75%	0.80%	0.85%	0.90%	0.95%	1.00%
0.02	0%	0%	0%	1%	1%	1%	1%	1%	1%	2%	2%	2%	2%	2%	2%	3%	3%	3%	3%	3%	3%
0.04	0%	0%	1%	1%	1%	2%	2%	2%	3%	3%	3%	4%	4%	5%	5%	5%	6%	6%	6%	7%	7%
0.06	0%	1%	1%	2%	2%	3%	3%	4%	4%	5%	5%	6%	6%	7%	8%	8%	9%	9%	10%	11%	11%
0.08	0%	1%	1%	2%	3%	3%	4%	5%	6%	6%	7%	8%	9%	10%	10%	11%	12%	13%	14%	15%	15%
0.1	0%	1%	2%	3%	3%	4%	5%	6%	7%	8%	9%	10%	11%	12%	13%	14%	15%	17%	18%	19%	20%
0.12	0%	1%	2%	3%	4%	5%	6%	8%	9%	10%	11%	12%	14%	15%	16%	18%	19%	21%	22%	24%	25%
0.14	0%	1%	2%	4%	5%	6%	8%	9%	10%	12%	13%	15%	16%	18%	20%	21%	23%	25%	27%	29%	31%
0.16	0%	1%	3%	4%	6%	7%	9%	10%	12%	14%	15%	17%	19%	21%	23%	25%	27%	29%	32%	34%	37%
0.18	0%	2%	3%	5%	6%	8%	10%	12%	14%	16%	18%	20%	22%	24%	27%	29%	32%	34%	37%	40%	43%
0.2	0%	2%	3%	5%	7%	9%	11%	13%	15%	18%	20%	23%	25%	28%	31%	33%	37%	40%	43%	47%	50%
0.22	0%	2%	4%	6%	8%	10%	12%	15%	17%	20%	23%	25%	28%	31%	35%	38%	42%	46%	50%	54%	58%
0.24	0%	2%	4%	6%	9%	11%	14%	16%	19%	22%	25%	28%	32%	35%	39%	43%	47%	52%	57%	62%	67%
0.26	0%	2%	5%	7%	10%	12%	15%	18%	21%	24%	28%	31%	35%	39%	44%	48%	53%	59%	64%	70%	77%
0.28	0%	2%	5%	8%	10%	13%	16%	20%	23%	27%	31%	35%	39%	44%	49%	54%	60%	66%	73%	80%	88%
0.3	1%	3%	5%	8%	11%	14%	18%	21%	25%	29%	33%	38%	43%	48%	54%	60%	67%	74%	82%	91%	101%
0.32	1%	3%	6%	9%	12%	15%	19%	23%	27%	32%	37%	42%	47%	53%	60%	67%	75%	83%	93%	103%	115%
0.34	1%	3%	6%	9%	13%	17%	21%	25%	29%	34%	40%	46%	52%	59%	66%	74%	83%	94%	105%	117%	132%
0.36	1%	3%	6%	10%	14%	18%	22%	27%	32%	37%	43%	50%	57%	64%	73%	82%	93%	105%	118%	134%	151%
0.38	1%	3%	7%	11%	15%	19%	24%	29%	34%	40%	47%	54%	62%	70%	80%	91%	103%	117%	134%	152%	174%
0.4	1%	3%	7%	11%	15%	20%	25%	31%	37%	43%	50%	58%	67%	77%	88%	101%	115%	132%	151%	174%	202%
0.42	1%	4%	8%	12%	16%	21%	27%	33%	39%	46%	54%	63%	73%	84%	97%	111%	128%	148%	172%	201%	236%
0.44	1%	4%	8%	12%	17%	23%	28%	35%	42%	50%	58%	68%	79%	92%	106%	123%	143%	167%	196%	232%	279%
0.46	1%	4%	8%	13%	18%	24%	30%	37%	44%	53%	63%	73%	86%	100%	117%	136%	160%	189%	225%	272%	333%
0.48	1%	4%	9%	14%	19%	25%	32%	39%	47%	57%	67%	79%	93%	109%	128%	151%	179%	215%	260%	321%	407%
0.5	1%	4%	9%	14%	20%	26%	33%	41%	50%	60%	72%	85%	101%	119%	141%	168%	202%	246%	304%	386%	511%
