# Supplementary material for: A broader lens on tuberculosis cost-effectiveness analysis: How patient-incurred costs and post-tuberculosis outcomes reshape estimates in a multi-country study
Source: PLOS Glob Public Health. 2025 Sep 11;5(9):e0005062. doi: 10.1371/journal.pgph.0005062 (PMC12425205; doi:10.1371/journal.pgph.0005062)
Supplement: Attachment — Submitted filename: response_to_reviewers.docx [file pgph.0005062.s008.docx]

| Final deadline for revised manuscript 5^th^ Jun 2025 | |
| --- | --- |
| **Row no.** | **Comments and responses** |
|  | For clarity, any text quoted from the originally submitted manuscript is given in “green quotes”.  Updated text, as it now appears in the revised manuscript, is given in “blue quotes”.  Numbers given in right hand column are consistent with those provided by the reviewers. (i-iii have been added for clarity under the second reviewer’s overall comments) |
|  | **Reviewer 1** |
| **Overall** | **Comment:** This is an excellent, and highly important, paper. My congratulations to the authors who have developed a very nice way of demonstrating and articulating the importance of extending CEA timeframes and perspectives. The paper is very well-written overall, and I only have a few comments - most of which are minor. |
|  | **Response:** We are very grateful for the positive and constructive feedback. We appreciate your assessment of the paper’s contribution and thank you for your time in reviewing it and for the thoughtful suggestions to strengthen it further. |
| **1** | **Comment:** You don’t currently explain how you estimate health benefit from vaccines in any detail. Can you expand section 2.3.4 to explain in further detail how vaccines were modelled, including any targeting or transmission impacts. |
|  | **Response:** We agree that additional clarity on this point is important. We’ve expanded Section 2.3.4 to describe the vaccine modelling approach in more detail.  Where we previously stated in Section 2.2 of the methods “Our analyses did not account for disease transmission”, this has been expanded to now read:  “Our analyses focused on the direct health effects of the intervention—defined as reductions in TB incidence, mortality (captured as years of life lost, YLL), and morbidity (captured as years lived with disability, YLD, using disability weights for active TB and post-TB health states). We did not model transmission dynamics or indirect (herd) protection effects. This simplification allowed for a consistent modelling approach across countries and enabling us to isolate the impact of key analytic choices under our different approaches”.  Section 2.3.4 - previous text:  “We assume values of 0.8 for both the intervention’s efficacy and coverage. [27, 28].”  Section 2.3.4 has been renamed from “Intervention Parameters” to now be  “Health outcomes and Intervention effect Modelling”  The revised text reads:  “We modelled a generic preventive intervention (e.g., a vaccine) applied uniformly across the entire population, without preferential targeting by age, risk group, or co-morbidity. Our core analysis assumed fixed values of 80% efficacy and 80% coverage. [27, 28] This resulted in a reduction in TB incidence across the population, applied as a scalar to all incident TB cases (i.e., incidence × [1 − efficacy × coverage], equivalent to a 64% reduction in our base case). This reduction then flowed through the model structure to reduce TB deaths, YLL, YLD, and DALYs, with all other epidemiological parameters held constant between intervention and comparator scenarios. We did not include disease transmission dynamics or indirect (herd) protection effects. This simplification allowed us to isolate the impact of analytic choices—specifically cost perspective, time-frame, and post-TB morbidity—on cost-effectiveness results across settings.”  Modelling equations have also been added to the appendix to provide additional clarity. |
| **2** | **Comment:** In the equations on page 5 and 6, it might be useful to distinguish costs incurred by the health system during the TB episode and costs incurred in the post-TB period. This would make the equations match your Figure 1. |
|  | **Response:** Thanks for this suggestion – the equations in 2.2 have been amended accordingly to align with the framework provided in Figure 1. The term ∆C_hs,aTB_ is defined and used in place of ∆C_hs_.  We have additionally updated our text:  “The most basic evaluation conforms to the conventional time period under a health system perspective,  including health system costs during active TB (∆C_hs,aTB_)…” |
| **3** | **Comment:** “Assuming the sex- and age distribution within each country was unchanged from 2019, we took 5-year breakdown from the IHME Global Health Data Exchange.” – I don’t quite understand this sentence. 5-year breakdown of what? |
|  | **Response:** Thank you for highlighting the ambiguity here. We have revised the Methods section to clarify that we used sex-disaggregated, 5-year age group population data from IHME’s GBD 2019 to disaggregate the broader age-band incidence and mortality estimates reported by WHO (e.g., 0–14, 15–24, etc.). This allowed us to estimate TB burden in finer 5-year age-sex subgroups. The GBD 2019 dataset was the most recent publicly available source for these demographic distributions, and we assumed relative stability in population structure since 2019 across countries. The updated paragraph now explains this process in clearer terms (see Methods section 2.3.1).  The previous wording read:  “TB mortality and total incidence estimates for each country (2022) were downloaded from WHO databases;  [24] these data are provided in sex-disaggregated 10-year age brackets. Assuming the sex- and age-  distribution within each country was unchanged from 2019, we took 5-year breakdown from the IHME  Global Health Data Exchange (GHDx) [25]. We applied each country’s mortality and incidence brackets to these proportions to reach 5-year sex-age subgroup estimates.”  The new wording is as follows:  “TB incidence and mortality estimates for 2022 were obtained from WHO databases, which are provided disaggregated by sex and reported in broad age categories (e.g., 0–14, 15–24, 25-34) for each country. To convert these into more granular 5-year age groups, we used population distribution data from the Global Burden of Disease Study 2019 (IHME), which provides sex-disaggregated estimates in 5-year age bands (e.g., 0–4, 5–9, etc.). Assuming that age-sex demographic structures have remained stable since 2019, we applied these proportions to disaggregate the WHO estimates. This enabled us to estimate incidence and mortality for each 5-year sex-age subgroup, facilitating more detailed DALY calculations across demographic strata.” |
| **4** | **Comment:** It would be useful to be more specific about your source of cost data – it doesn’t look like the WHO report cited provides data specific enough for you to have included in your analysis? Did you receive the actual data from WHO? |
|  | **Response:** Thank you for raising this point.  We have provided additional detail in the manuscript relating to the handling of patient costs. Our analyses did not utilise any unpublished data from the WHO or otherwise.    It is important however for us to provide additional clarity relating to which costs were based on empirical estimates, and which were modelled within our study. This clarity is provided below, and has been added to the manuscript.  **Patient costs:**  All patient cost inputs used in our analysis were extracted from the WHO’s publicly accessible *“National surveys of costs faced by tuberculosis patients and their households 2015-2021”,* which summarises patient cost findings from 20 TB patient cost studies. This can be downloaded [[HERE](https://iris.who.int/bitstream/handle/10665/366277/9789240065536-eng.pdf?sequence=1)] and is our reference [3] in the manuscript. These costs are reproduced in our Table 1 for 19 countries (we did not include Zimbabwe in our analysis). Costs were applied for MDR-TB and DS-TB separately, other than for the two countries which had not provided disaggregated costs in their surveys (Fiji and Solomon Islands), for which overall costs were used for all patients undergoing treatment. We applied patient costs only to those who underwent treatment in our model, determined by the case detection rate for each country, with rates provided by the WHO.  *Patient costs in the period up to treatment completion:*  For the period up until treatment completion, we applied the cost as provided in Table 1. That is, a patient in Kenya undergoing DS-TB treatment would incur a cost of $138 for DS-TB treatment, or $803 for MDR-TB treatment.  *Patient costs (in the post-TB period)*  For the post-TB period, the Extended time-frame under the societal perspective (providing the so termed ‘Enhanced ICER’), we applied post-TB costs as described in section 2.3.6 ‘Patient costs’ and further outlined in the Appendix on page 33, “Lost Income Post-Treatment Completion”. This was based on the reported monthly income before TB and that during treatment.  The wording in 2.3.6 has been amended slightly to add clarity, and the following sentences have been added:  “No additional patient-level cost data were sourced beyond the WHO’s publicly available surveys; all values used are drawn directly from this dataset and adjusted as described. These are presented in Table 1 and Table A4 (Appendix).”  **Health System costs –**  No unpublished WHO data were used in our cost estimation. As described in Section 2.3.5, our approach focuses on estimating societal (provider + patient) costs by leveraging model outputs directly. Therefore, detailed health system cost breakdowns (e.g., individual unit costs for diagnostics or medication) were not needed, and we did not source separate HS cost tables — these are absorbed into aggregate cost estimates based on the model structure. |
| **5** | **Comment:** I realise that this is just an instrumental model, so properly managing uncertainty may not be necessary, but it would be useful to mention this in your methods/discussion. Some of the WHO estimates have very wide error bars – how would this uncertainty affect your findings? |
|  | **Response:** We agree that this is an important caveat. We have recognised this limitation in the limitation section of our Discussion as suggested:  “Moreover, several of the WHO-derived parameters—such as TB incidence, case fatality rates, and detection rates—have considerable uncertainty, often provided with wide confidence intervals. While our model is instrumental, this uncertainty could influence the scale of our findings, particularly for country-level estimates, and should be borne in mind.” |
| **6** | **Comment:** Do you have anything to justify your assumption of a linear return to pre-TB income, and the time frame for return? Also, In the text you say the range is 1-10 years, and in Table 2 this is listed as 0-10 years. |
|  | **Response:** Thank you for raising this point. While we’re not aware of studies that follow patients until their income fully returns to pre-TB levels, a small number of longitudinal studies do provide insight into the duration and extent of economic recovery post-treatment. We have now referenced these studies in the manuscript and clarified our assumptions. Additionally, we have revised the wording in the limitations section of the Discussion to better reflect the uncertainty surrounding this parameter.  The text in our limitations now reads:  “Second, our analysis was constrained by a lack of long-term data on both patient and health system post-TB costs. While we assumed in our base-case analyses that household finances recover approximately three years after TB treatment completion, this is based on limited empirical evidence and likely varies by setting. [6–8,29]”  Section 2.3.6 of our Methods, revised to provide greater clarity on this issue, now reads:  “We extracted average household income before and during TB from the patient cost survey data  and assumed a linear return to pre-TB income levels following treatment completion. This  simplifying assumption was informed by cohort studies from Malawi, India, and Brazil, which found  that income and employment often remain suppressed up to 12 months post-treatment, with only partial  recovery by two to three years. [6–8,29] This return period was assumed to be 3-years in core analyses  (varied from 1 to 10 years in sensitivity analyses), with a 3% annual applying a discount  rate of 3% applied.”  Thank you also for spotting the inconsistency and for raising this point regarding the range. We’ve now clarified that the base case assumes a linear income return over a 3-year period, varied between 1–10 years in sensitivity analysis. |
| **7** | **Comment:** In Table 1, can you label whether the cost data are presented in 2022 USD or in the year of study |
|  | **Response:** Thank you for raising this point. This clarity has been added to Table 1. Costs are all adjusted using the stated inflation factor to 2022 USD. The table footnote and column headings have been revised for clarity:  *Previous footnote:*  † The inflation factor indicates the multiplier used to adjust costs collected in year of survey (study year) to 2022 prices.  *Revised footnote:*  † All cost values in this table have been adjusted to constant 2022 US dollars using the listed inflation factor.  *Changes to column headings in Table 1:*  “Overall Patient Costs” ⟶ “Overall Patient Costs (2022 USD)”  “First-line (FL) Patient Costs” ⟶ “First-line (FL) Patient Costs (2022 USD)”  “MDR-TB Patient Costs” ⟶ “MDR-TB Patient Costs (2022 USD)” |
| **8** | **Comment:** Section 3.1.1 – it might be worth testing correlation of direct and indirect costs separately. |
|  | **Response:** We appreciate this suggestion – these analyses have been conducted and added to the appendix.  We have added the following text, explaining how no correlation was found, and have included a new Figure A3 in our appendix to demonstrate this graphically (see below).    “Pearson correlation found no significant relationship between GDP per capita and either DSTB patient costs (*p*=0.534) or MDR-TB costs (*p*=0.927). (See Figure A2 Appendix for graphical illustration).” “We also  found no significant relationship between GDP per capita and individual cost components overall: Direct  Medical (*p*=0.830), Direct Non-medical (*p*=0.920), and Indirect costs (*p*=0.146) (see Appendix Figure  A3).”    Note that the WHO’s *“National surveys of costs faced by tuberculosis patients and their households 2015–2021”* provides the patient cost breakdown for “Overall Costs” only and does not present disaggregated data for MDR-TB and DS-TB separately. As all the patient cost data we referenced were taken directly from this summary document, we did not extract individual cost values from the original country-level reports. We believe this approach is consistent with and appropriate for the purpose of our analysis, which aimed to use standardised, comparable data across settings. |
|  | **Reviewer 2** |
| **Overall** | **Comment:** The paper is well written and addresses important concepts of perspectives and timeframes adopted when conducting cost-effective analysis. The authors make a valuable contribution by investigating their impact on TB ICERs. Their results justify using more detailed approaches in the economic evaluations of TB. |
|  | **Response:** We are again grateful for the comments and points raised. We’re pleased the study’s contribution was clear, and we’ve made the following revisions in response to your helpful feedback. |
| **i** | **Comment:** A limitation of the study is the exclusion of the healthcare costs associated with long-term disability. It is justified in figure 1’s footnote, but it is an important point and should be mentioned in the methods section, and its implications on the results should be more expanded on in the text. |
|  | **Response:** We’ve now added text in the Methods to more clearly acknowledge that long-term healthcare costs due to disability are excluded. The following text has been added to section 2.2 after describing the approaches:  “Post-TB health system costs—such as those related to managing long-term disability—were excluded from all approaches in the core analysis due to a lack of robust data across settings. However, their potential impact was explored through a separate scenario analysis (see Section 2.4.3).”  In the Discussion, we note that including even modest long-term disability management costs would raise the ICERs (we explored a range of post-TB care costs in a scenario analysis to quantify this)  “Similarly, post-TB healthcare costs remain poorly documented, and our analysis does not account for the financial burden of managing long-term TB-related complications, which could influence cost-effectiveness estimates if systematically included.” |
| **ii** | **Comment:** In 4.1, paragraph 2, p.22, there is a very good point made about the weight on patient costs in wealthier settings and the country’s GDP. Did you try grouping the countries by income level and checking how the results compare? |
|  | **Response:** Thank you for this suggestion.  We ran an analysis grouping the countries according to the World Bank’s income classification (low-, lower-middle-, and upper-middle-income) and calculated the average percentage change in ICERs when patient-incurred costs are excluded. This analysis uses the societal perspective under the conventional timeframe, which best isolates the marginal effect of omitting patient-incurred costs. We found that excluding patient-incurred costs increased ICERs by an average of 12.6% in low-income countries, 11.3% in lower-middle-income countries, and 5.9% in upper-middle-income countries.  These results support our observation that although absolute patient costs remain high across settings, their proportional influence on ICERs is lower in countries with higher GDP-based cost-effectiveness thresholds. We have added this to our Discussion (Section 4.1) to strengthen the interpretation.  “Notably, we found no significant overall correlation between a country's GDP and the magnitude of TB patient costs, yet several countries with the highest average patient cost burdens (e.g., Laos, Ghana, Nigeria) also have relatively low GDP per capita, likely due to weaker health financing arrangements and social protection systems leading to higher out-of-pocket expenses. This highlights an important issue, being that analyses using GDP-based thresholds may place less weight on patient costs in wealthier settings, potentially obscuring the true financial burden of TB, especially in middle-income countries where patient costs remain high but appear less influential proportionally. When we examined results in relative terms (percent change in ICER), patient-incurred costs were still a substantial component across all settings, suggesting that GDP-based ICER thresholds can mask the impact of these costs rather than eliminate it.” “Grouping countries by World Bank income classification, we found that excluding patient-incurred costs increased ICERs by an average of 12.6% in low-income, 11.3% in lower-middle-income, and 5.9% in upper-middle-income countries, supporting the observation that the relative influence of patient-incurred costs on cost-effectiveness estimates diminishes in higher-income settings with larger GDP-based thresholds.” |
| **iii** | **Comment:** More details on the costs included in the patients incurred cost would help understand what is taking into account and put the results into perspective. |
|  | **Response:** Thank you, please see our response to comment 4 from Reviewer 1 above.  Patient-incurred costs in our analysis comprise all out-of-pocket expenditures and income losses incurred by TB patients and their households during treatment, as reported in the WHO survey data (see Table 1 for country-specific values). We did not include additional costs beyond treatment completion, aside from the lost income due to post-TB morbidity (modelled via the 1–10 year income recovery assumption), due to the scarcity of longer term cost data. |
|  | **Minor** |
| **1** | **Comment:** In the title, it might be better to avoid using abbreviations and write tuberculosis instead of TB. |
|  | **Response:** We’ve now revised the title to spell out “tuberculosis” in full.  Revised title:  “A Broader Lens on Tuberculosis Cost-Effectiveness Analysis:  How Patient-Incurred Costs and Post-tuberculosis Outcomes Reshape Estimates in a Multi-Country Study” |
| **2** | **Comment:** In the introduction, p.3, “The societal perspective, rooted in welfare economics, [12] aims to maximise gains for society as a whole, incorporating all relevant costs and consequences, irrespective of who incurs them. While theoretically broad, its real-world application is often constrained, though the inclusion of patient costs is expected.” I find the last sentence a little bit confusing. Maybe if we add a sentence to explain more why it is constrained and a link to the last part of the sentence. |
|  | **Response:** Thank you for flagging this. We’ve revised the sentence to provide additional clarity.  The new sentence reads:  “Grounded in welfare economics [12], the societal perspective aims to capture the full economic impact of a health intervention across all members of society by incorporating all relevant costs and consequences, irrespective of who incurs them, and to maximise overall societal benefit. Despite its conceptual breadth, in practice analysts often face challenges in applying the full societal perspective due to limited data on non-health sector costs, such as productivity losses or informal caregiving time. Nevertheless, inclusion of at least some patient-incurred costs—such as direct payments and lost income—is widely considered a minimum expectation when adopting this perspective.” |
| **3** | **Comment:** Some abbreviations are in capital letters and some are not (ds-TB in the last paragraph p.12, dalys last paragraph p.18), it would be good to be consistent. Also, SAE is not defined the first time it is used in the manuscript. |
|  | **Response:** We’ve conducted a full check to ensure consistency in abbreviation formatting and have defined SAE as ‘serious adverse event’ at first use. Thank you for raising this. |
| **4** | **Comment:** Last paragraph p.12, lifetime costs were varied between $1 and $100. It would be good to mention what informed this range. |
|  | **Response:** Thanks for raising this – we understand the need for transparency here. We have added some additional information in section 2.4.3 following the sentence reading: “We explored the impact on the Enhanced ICER for a wide range of lifetime costs, from $1 to $100 dollars per person post-TB.”  The added sentence reads:  “This range ($1–$100) was selected to reflect a plausible lower- and upper-bound scenario, given the absence of robust data on post-TB health service utilisation. It draws on limited empirical studies suggesting ongoing disability in a proportion of patients, but acknowledges the wide uncertainty in actual treatment costs.”  We have additionally added some text in our Limitations section where this is discussed.  See old text (green) and new text (blue) below.  “Second, our analysis was constrained by a lack of long-term data on both patient and health system post-TB costs. While we assumed household _nances recover approximately three years after TB treatment completion, this is based on limited evidence and may vary significantly by setting. Similarly, post-TB healthcare costs remain poorly documented, and our analysis does not account for the financial burden of managing long-term TB-related complications, which could influence cost-effectiveness estimates if systematically included.” “Given this uncertainty, we applied a wide ($1–$100) range in sensitivity analyses to explore plausible scenarios.” |
| **5** | **Comment:** Last sentence p.16: the average effect of excluding post-TB effects on ICERs is quantified, but that of omitting patient-incurred costs is not here. It might be good to have similar numbers (average and range) for this part as well. |
|  | **Response:** Thanks for raising this. The text below the table has been updated as follows:  Old text:  “Across all countries, excluding post-TB effects increased ICERs by an average of 51% (ranging from +19.3% in Ghana to +108% in Mongolia), while omitting patient-incurred costs had a smaller effect, except in the lowest-GDP setting.”  New text:  “Across all countries, excluding post-TB effects increased ICERs by an average of 51% (ranging from +19.3% in Ghana to +108% in Mongolia), while omitting patient-incurred costs had a smaller effect, increasing ICERs by an average of 14% (from +0.6% in Papua New Guinea up to +54.4% in Mali).” |
| **6** | **Comment:** Discussion: the discussion is well structured, and very interesting points were addressed. |
|  | **Response:** We appreciate this feedback and are glad the Discussion was useful. |
| **6**a | **Comment:** In the 2nd paragraph, “The average increase in ICERs across countries was 51%, highlighting the need for greater consideration of post-TB health impacts in cost-effectiveness analyses, particularly for preventative interventions.”, can you elaborate on why it is particularly for preventative interventions. |
|  | **Response:** Thank you — we agree this point warrants clarification. While post-TB health outcomes are important to consider across all TB interventions, their omission carries particularly significant implications for preventative strategies. Preventing TB entirely ensures that any post-TB sequelae that would have arisen are fully averted. By contrast, interventions delivered at the point of diagnosis or treatment provide less certainty in avoiding these long-term consequences.  The new text to replace the text quoted reads:  “The average increase in ICERs across countries was 51%, highlighting the need for greater consideration of post-TB health impacts in cost-effectiveness analyses. While relevant across all intervention types, these impacts are particularly salient for preventative strategies, where TB incidence is averted entirely and with it, all downstream morbidity and disability. |
| **6b** | **Comment:** In the 3rd paragraph, the cost-effectiveness threshold is 2*GDP. It might be good to add a sentence and explain why it is different from the 1*GDP in the 2nd paragraph. |
|  | **Response:** Thank you for this comment. The reference to 2× GDP per capita in the third paragraph more reflects an empirical finding than a decision-making threshold. As shown in Figure A4, the relative difference between our extended and conventional ICERs tends to stabilise by this point, suggesting that the ratio becomes less sensitive to further increases in the willingness-to-pay threshold. We have revised the text to clarify this, as follows:  Previous text:  “By contrast, including patient costs had a smaller effect on ICERs. For almost all countries, at cost-effectiveness thresholds of 2*GDP per capita or higher, patient costs had minimal impact. The largest influence was observed in countries where patient costs were high relative to GDP per capita, such as Mali and Uganda, where ICERs were 20% and 11% lower under the societal perspective, respectively.”  Updated text:  “By contrast, including patient costs had a relatively limited effect on ICERs (as estimated using our extended approach). In all countries, their influence diminished as the cost-effectiveness threshold increased, with the relative difference between ICERs (under the societal *vs.* health system perspectives) plateauing or changing minimally beyond approximately 2× GDP per capita (Figure A4). At these higher thresholds, patient costs contributed little to the overall ICER. The largest impacts were seen in countries where patient costs were especially high relative to GDP, such as Mali and Uganda, where ICERs were 20% and 11% lower, respectively, under the societal perspective.” |
| **6c** | **Comment:** Point 4.1, 3rd paragraph: “Additionally, GDP-based thresholds cannot fully account for the pricing structure of interventions such as vaccines, which are often globally standardised rather than varying proportionally with GDP”, the second part of the sentence is not very accurate as vaccine prices are not globally standardised. BCG is very cheap across countries, but the sentence seems broader than BCG. |
|  | **Response:** Thank you for this helpful clarification. We acknowledge that the original phrasing may have overstated the degree of global standardisation in vaccine pricing. To improve accuracy, we have revised the sentence as follows:  “Additionally, GDP-based thresholds cannot necessarily account for the pricing structure of some interventions, such as vaccines, which may be subject to tiered pricing or global procurement mechanisms that may not align closely with national income levels.” |
